# A Novel Vav3 Homolog Identified in Lamprey, *Lampetra japonica*, with Roles in Lipopolysaccharide-Mediated Immune Response

**DOI:** 10.3390/ijms18102035

**Published:** 2017-09-22

**Authors:** Yanqi Shen, Yishan Zhang, Yinglun Han, Peng Su, Meng Gou, Yue Pang, Qingwei Li, Xin Liu

**Affiliations:** 1 College of Life Science, Liaoning Normal University, Dalian 116081, China; lsdshenyq@126.com (Y.S.); lnnuyshzhang@126.com (Y.Z.); hanyinglun@163.com (Y.H.); sp4046@163.com (P.S.); gouer602@126.com (M.G.); pangyue01@163.com (Y.P.); 2 Lamprey Research Center, Liaoning Normal University, Dalian 116081, China

**Keywords:** *Lampetra japonica*, Vav3, Phylogenetic relationship, Expression pattern, LPS-mediated immune response

## Abstract

Vav guanine nucleotide exchange factor 3 (Vav3), a Rho family GTPase, regulates multiple cell signaling pathways including those of T- and B-cell receptors in vertebrates through mediating the activities of the Rho family members. Whether the lamprey possesses Vav3 homolog and what role it plays in immune response remain unknown. Gene cloning, recombinant expression, antibody production and expression pattern analyses were performed to characterize the lamprey Vav3 in the current study. The lamprey Vav3 is closer to jawed vertebrates’ Vav3 molecules (about 53% identities in general) than to Vav2 molecules of jawless and jawed vertebrates (about 51% identities in general) in sequence similarity. Conserved motif analysis showed that the most distinguished parts between Vav3 and Vav2 proteins are their two Src-homology 3 domains. The relative expression levels of lamprey *vav3* mRNA and protein were significantly up-regulated in lamprey lymphocytes and supraneural myeloid bodies after mixed-antigens stimulation, respectively. In addition, lamprey Vav3 were up-regulated drastically in lymphocytes and supraneural myeloid bodies after lipopolysaccharide (LPS) rather than phytohemagglutinin (PHA) stimulation. Lamprey Vav3 distributed in the cytoplasm of variable lymphocyte receptor B positive (VLRB^+^) lymphocytes, and the number of plasmacytes (VLRB and lamprey Vav3 double positive) in blood lymphocytes also increased after LPS stimulation. Our results proved that lamprey Vav3 was involved in the LPS-mediated immune reaction of lamprey and provided a clue for the further study of the precise role lamprey Vav3 played in the signaling pathway of lamprey VLRB^+^ lymphocytes.

## 1. Introduction

Vav family proteins are a group of guanosine nucleotide exchange factors that contain Dbl homology (DH) domains and possess catalytic activity specifically directed towards Rho- and Rac-GTPases. In mammals, it is known that there are three members in Vav protein family, Vav1, Vav2 and Vav3 [1]. Although Vav1, Vav2 and Vav3 have identical structural domains, they are different in their tissue distributions. Vav1 distributes specifically in hematopoietic system cells, while Vav2 and Vav3 have broader patterns of expression [2,3]. Vav family members contain eight conserved domains: calponin-homology (CH), acidic (Ac), Dbl-homology (DH), pleckstrin-homology (PH), zinc finger (ZF), N-terminal Src-homology 3 (NSH3), and Src-homology 2 (SH2) and C-terminal SH3 (CSH3) domains from N- to C-terminals [2,4,5,6,7]. The CH domain is proven to be involved in both the regulation of Vav guanosine nucleotide exchange factor activity and the pathway of Ca^2+^ mobilization. The Ac domain, which contains three highly conserved tyrosine residues (Y), is an auto-inhibition mediator of Vav guanosine nucleotide exchange factor activity. The DH domain of Vav3 can interact with Rac- and Rho-GTPases to promote GDP exchange to GTP. The PH domain is the interaction site for Vav3 interacting with GTP binding protein, lipid and the phosphorylated serine/threonine residues. The ZF domain, also known as C1 domain, is a cysteine rich domain, which is the key site for Vav3 binding to protein kinase C or diacylglycerol kinase. As a single functional unit, the NSH3 domain of Vav3 can facilitate the interaction with its protein partners [8,9]. The SH2 domain possesses high affinity with protein tyrosine phosphatase and is involved in the regulation of kinase activity and substrate phosphorylation [10]. The CSH3 domains of Vavs are included in binding their interactive proteins, and there are differences in the protein spectrum that they bind [11,12].

As a member of the Vav family, Vav3 has been studied extensively in recent years. Movila et al. [4] first found Vav3 by using Expressed Sequence Tags (EST) technique in human placenta cDNA library in 1999, and the *vav3* gene was found mapped on human chromosome region 1p13.3 [2]. Human *vav3* gene encodes a protein of 847 amino acids, which is also composed of the same conserved functional domains as Vav1 and Vav2 [4]. Vav3 is widely distributed in various tissues and plays important roles in the formation of cytoskeleton and cell differentiation, and also plays important roles in the regulation of T and B cell signaling pathways. The *vav3*-deficient B cells exhibit similar defects as the phosphatidylinositol 3-kinase (PI3K)-deficient B cells, suggesting that Vav3 and PI3K may have interactive function [13]. Vav3 was further proved to be a positive regulator of PI3K in the B cell receptor (BCR) signaling pathway and the up-regulation of PI3K activity is achieved by Ras-related C3 botulinum toxin substrate 1 (Rac1) in a GTP-dependent manner [14]. Vav3 can be rapidly phosphorylated after T cell receptor (TCR) activation, which requires SH2 domain-containing leukocyte phosphoprotein of 76 kDa (SLP-76) association for its membrane translocation, indicating that it can transduce signals from the receptor [15]. It was found that, if *vav3* is lacking or impaired, mature T cells proliferate poorly during T cell development, suggesting that Vav3 plays an important role in signal transduction pathways of TCR [16]. In recent years, some studies showed that Vav3 has the function of the proto-oncogene, and is involved in the formation of a variety of tumors [17,18].

Lampreys belong to the Cyclostomata, which comprises a group of the most primitive vertebrates. For a long time, lampreys have been regarded as key species for studying the evolution of vertebrates, which are ideal model animals in the research fields of comparative anatomy [19], developmental biology [20], ecology [21], immunology [22,23], etc. They were found not only to bear a number of primitive characteristics similar to the innate immune system of higher vertebrates, but also to exhibit immunological memory similar to adaptive immune system of higher vertebrates [22]. Instead of B cell receptor (BCR) and T cell receptor (TCR), which possess combinatorial diversity in the adaptive immune system of jawed vertebrates, variable lymphocyte receptor A (VLRA), VLRB, and VLRC were discovered successively in jawless vertebrates as the counterparts of the BCR and TCR to recognize the external pathogens [24,25,26]. In addition, VLRB positive (VLRB^+^) lymphocyte subset is mainly distributed in periphery blood, and it can proliferate and differentiate to plasmacyte-like cells that express VLRB tetramers or pentamers after pathogen stimulation, which is equivalent to immunoglobulin M expressed by plasmacytes [27,28]. VLRA^+^ and VLRC^+^ lymphocyte subsets are mainly distributed in lamprey “thymoid” gill region, and express their specific VLR molecules only on their cell-surface. VLRA^+^ and VLRC^+^ lymphocyte subsets also express orthologous genes which are used by αβ and γδ T cells of jawed vertebrates for their differentiation, respectively [26,29].

Compared with the deep understandings of the Vav3 functions in the adaptive immune system of jawed vertebrates, it is still a blank about the existence and function of *vav3* plays in the adaptive immune system of jawless vertebrates. Previously, we performed next-generation sequencing approach to explore transcriptomic responses of lamprey (*Lampetra japonica*) lymphocyte-like cells to immune-stimuli, and a Vav3 homologous sequence was found. In the present study, we report on the molecular cloning and characterization of the lamprey Vav3 for the first time.

## 2. Results

### 2.1. Identification of Lamprey Vav3 Sequence

**Figure 1:**
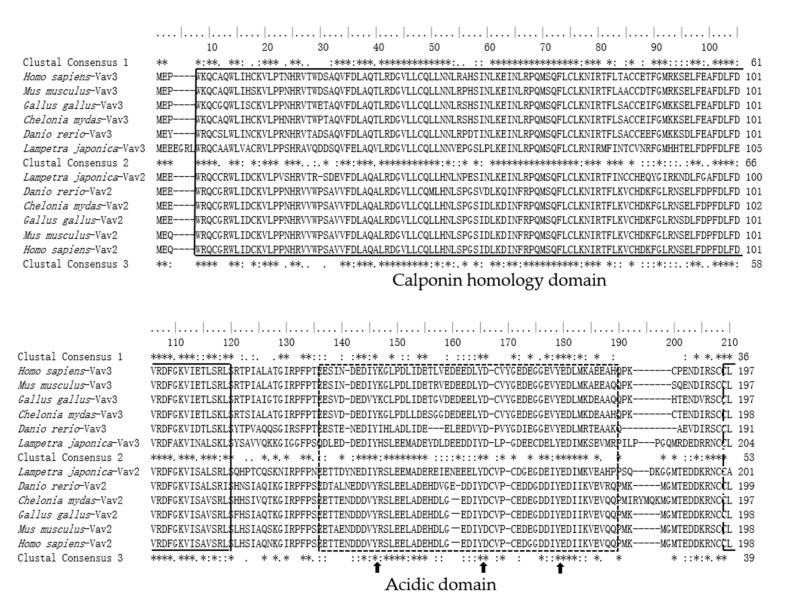

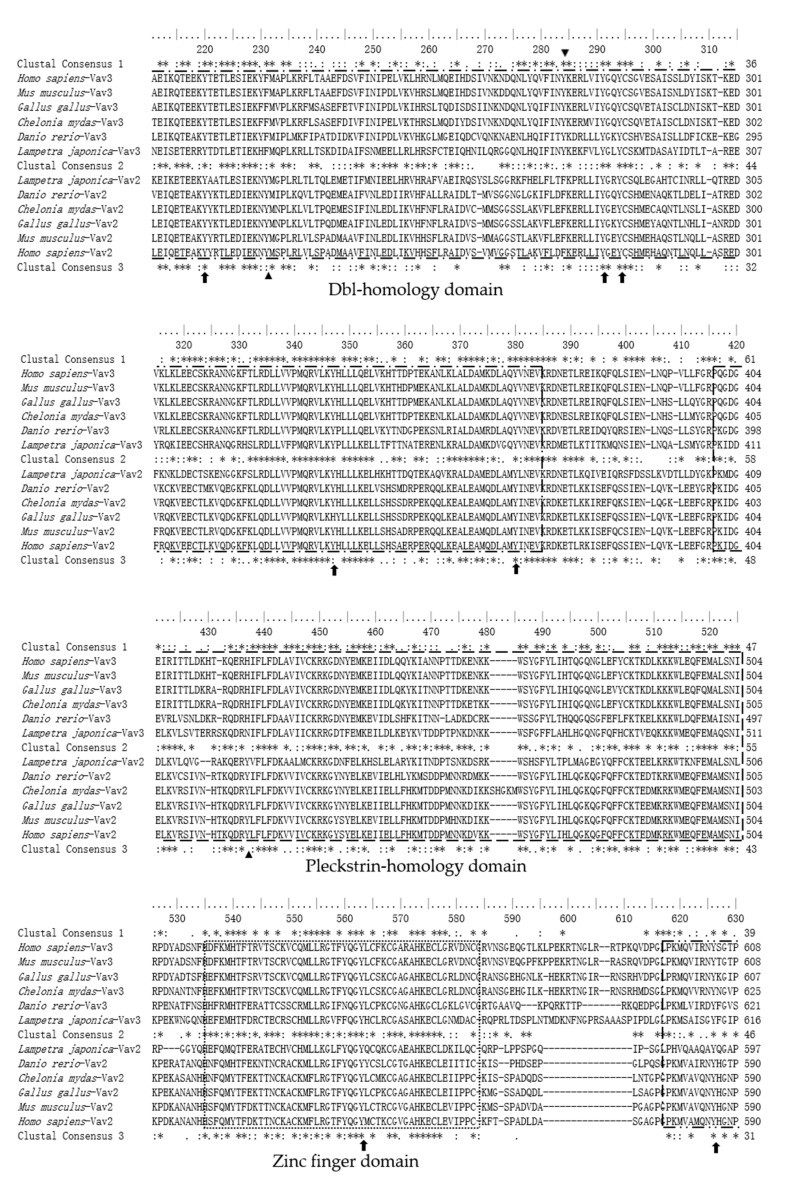

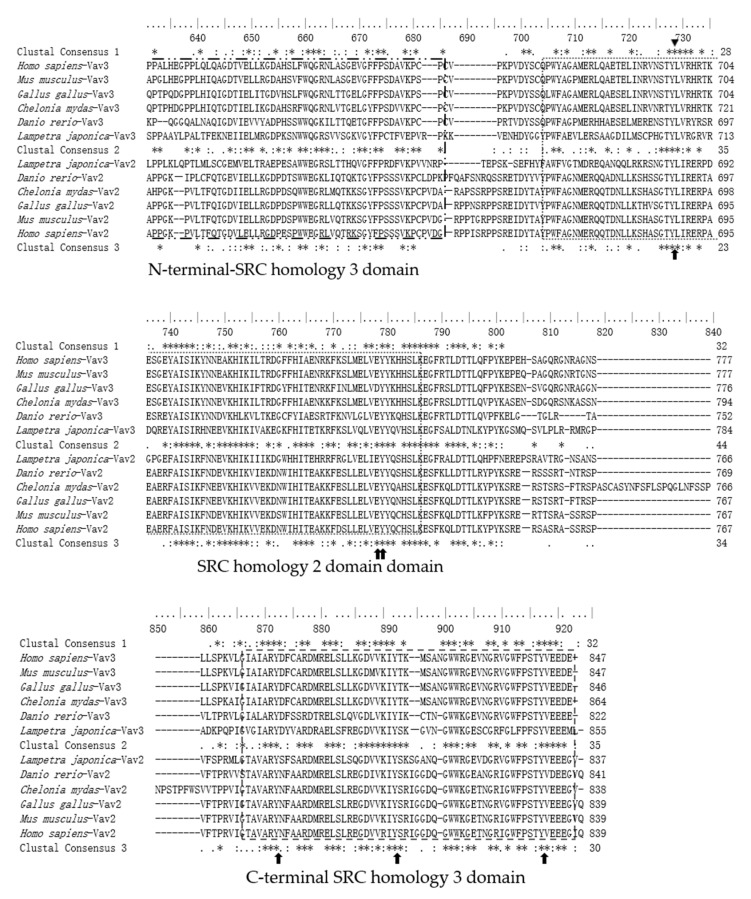
Multiple sequence alignment analyses of lamprey Vav3 protein sequence with several Vav3 and Vav2 molecules of jawed vertebrates. The accession numbers of these protein sequences are listed below. *Homo sapiens*-Vav3: AAD20349.1; *Mus musculus*-Vav3: NP_065251.2; *Gallus gallus*-Vav3: NP_996745.1; *Chelonia mydas*-Vav3: XP_007055271; *Danio rerio*-Vav3: NP_001119865.1; *Lampetra japonica*-Vav3: KX911208; *Lampetra japonica*-Vav2: gbAIN44441; *Danio rerio*-Vav2: XP_009293636.1; *Chelonia mydas*-Vav2: XP_007061074; *Gallus gallus*-Vav2: NP_989473.1; *Mus musculus*-Vav2: XP_006497921.1; and *Homo sapiens*-Vav2：NP_003362.2. The calponin homology (CH), acidic (Ac), Dbl-homology (DH), pleckstrin homology (PH), zinc finger (ZF), N-terminal SRC homology 3 (NSH3), SRC homology 2 (SH2) and C-terminal SH3 (CSH3) domains are marked with solid line, dashed line, long dash-dot line, long dashed line, dotted line, long dash-dot-dot line, double dashed line and double long dashed line, respectively. The conserved tyrosine residues between Vav3 and Vav2 molecules are indicated by arrows. The conserved tyrosine residues specific to Vav3 or Vav2 are marked by triangles and inverted triangles, respectively. The Clustal Consensus 1, 2 and 3 indicate the identical (*), highly homologous (:) and homologous (.) residues between lamprey Vav3 and Vav3 molecules of jawed vertebrates, between lamprey Vav3 and lamprey Vav2, or between lamprey Vav3 and Vav2 molecules of jawed vertebrates, respectively.

A cDNA fragment of lamprey *vav3* was amplified by PCR method from peripheral lymphocytes cDNA library of *L. japonica*. The open reading frame (ORF) of lamprey *vav3* is 2568-bp in length and encodes a protein containing 855 amino-acid residues with a theoretical molecular weight of 94.1-kDa. The lamprey Vav3 sequence was submitted to the GenBank database with an accession number of KX911208. By searching the combination of specific domains with online tool SMART, it was found that lamprey Vav3 also has the same combination of eight domains that are structure characteristics of Vav family, namely CH, Ac, DH, PH, ZF, NSH3, SH2 and CSH3 domains from N- to C-terminal. The sequence alignment result revealed that lamprey Vav3 possesses about 53% identity in general with Vav3 molecules of jawed vertebrates, but shares only 51.5% identity with a lamprey Vav2 (EMBL:AIN44441.1) and about 51% identity in general with Vav2 molecules of jawed vertebrate, respectively (Figure 1). In addition to the high sequence similarity between lamprey Vav3 and Vav3 molecules, lamprey Vav3 also shares two tyrosine residues (Y278 and Y716) that are only conserved among Vav3 sequences of jawed vertebrates. Considering there are also only two partial sequences that are homologous to Vav2 (Transcript: ENSPMAT00000002100.1) and Vav3 (Transcript: ENSPMAT00000002350.1), respectively, in the genome of *Petromyzon marinus*, it means that probably only two Vav family members, lamprey Vav2 and lamprey Vav3, exist in jawless vertebrates.

### 2.2. Phylogenetic Analysis of the Vav Family

**Figure 2:**
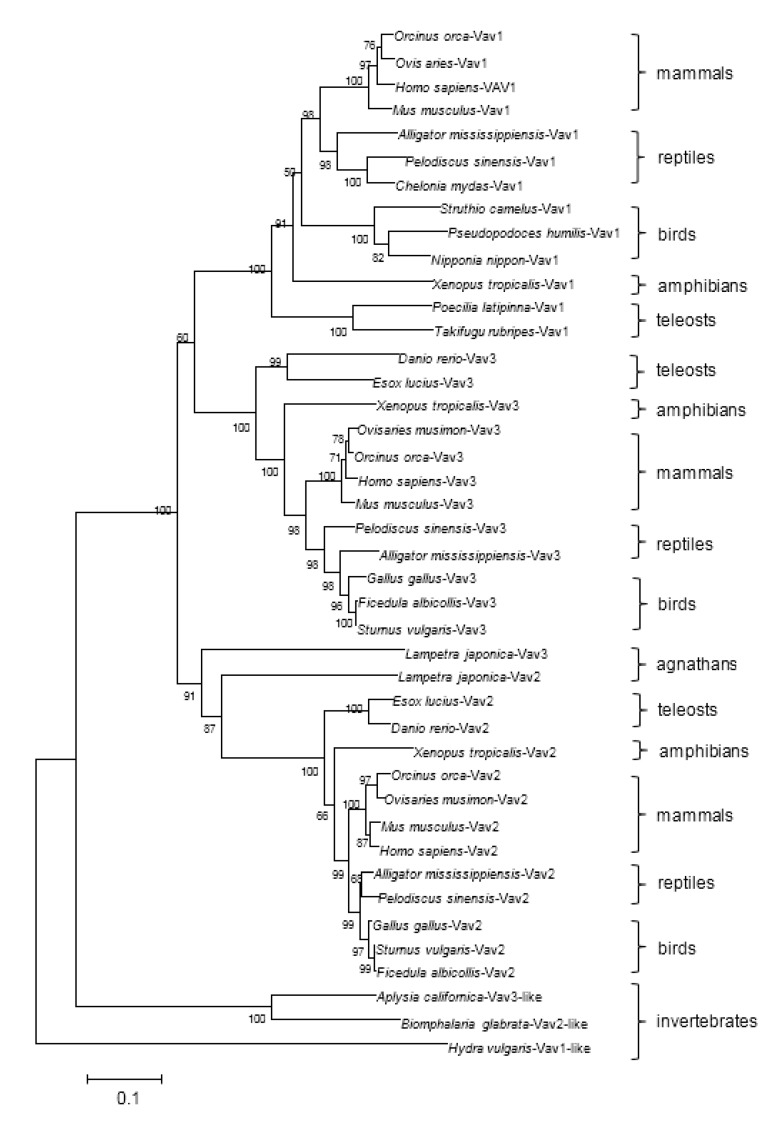
Phylogenetic analysis of lamprey Vav3 with Vav3, Vav2 and Vav1 homologs based on the neighbor-joining (NJ) method. The number at each node presents the percentage of bootstrapping after 1000 replications. Vav3, Vav2 and Vav1 homologs from lampreys, teleosts, amphibians, reptiles, birds, mammals and invertebrates are marked on the right side. The genetic distance is indicated by the ruler below the tree. The protein accession numbers not listed in Figure 1 are as follows: *Ovisaries musimon*-Vav3: XP_011979369; *Orcinus orca*-Vav3: XP_004263156; *Ficedula albicollis*-Vav3: XP_016155749; *Pelodiscus sinensis*-Vav3: XP_006119853; *Xenopus tropicalis*-vav3: XP_012817964; *Esox lucius*-Vav3: XP_010889475; *Sturnus vulgaris*-Vav3: XP_014740857.1; *Aplysia californica*-Vav3-like: XP_012934911; *Biomphalaria glabrata*-Vav2-like: XP_013065355; *Esox lucius*-Vav2: XP_010892236; *Xenopus tropicalis*-Vav2: NP_001039156; *Pelodiscus sinensis*-Vav2: XP_006123811; *Ficedula albicollis*-Vav2: XP_016157973; *Orcinus orca*-Vav2: XP_004285057; *Ovisaries musimon*-Vav2: XP_011973373; *Sturnus vulgaris*-Vav2: XP_014728763; *Orcinus orca*-Vav1: XP_004277328; *Ovis aries*-Vav1: XP_012034581; *Homo sapiens*-Vav1: NP_005419; *Mus musculus*-Vav1: NP_035821; *Struthio camelus*-vav1: XP_009668692; *Pseudopodoces humilis*-vav1: XP_014117547; *Nipponia nippon-*Vav1: XP_009465925; *Alligator mississippiensis*-Vav1: XP_006265667; *Pelodiscus sinensis*-Vav1: XP_006131759; *Chelonia mydas*-Vav1: XP_007070109; *Xenopus tropicalis*-Vav1: XP_002940062; *Poecilia latipinna*-Vav1: XP_014889433; *Takifugu rubripes*-Vav1: XP_011611118; and *Hydra vulgaris*-Vav1-like: CDG71338.

A Neighbor-Joining phylogenetic tree was reconstructed based on 42 sequences of Vav1 (-like), Vav2 (-like) and Vav3 (-like) by using the Clustal X and MEGA4 programs (Figure 2). The homologous sequences identified in invertebrates such as *Hydra vulgaris*-Vav1-like, *Biomphalaria glabrata*-Vav2-like and *Aplysia californica*-Vav3-like were used as the out-group. Phylogenetic analysis showed that Vav1, Vav2 and Vav3 sequences from fishes to mammals were mainly grouped into three big clusters, respectively. In Figure 1, it can be deduced that the first split of Vav family members happened between the Vav3 ancestor and the common ancestor of Vav1 and Vav2, and that the second split happened between the ancestors of Vav1 and Vav2, which probably derived from gene duplication events.

Lamprey Vav3 is grouped together with lamprey Vav2 as two single branches in the cluster of Vav2 sequences, and the genetic distance between lamprey Vav3 and the common ancestor of Vav2 molecules and lamprey Vav2 is closer than that between lamprey Vav3 and the ancestor of Vav3 molecules. These results indicate that lamprey Vav3 might originate from a common ancestor who was close to Vav2 in genetic distance through a gene duplication process.

### 2.3. The Sequence Differences between Vav3 and Vav2 Subfamily

**Figure 3:**
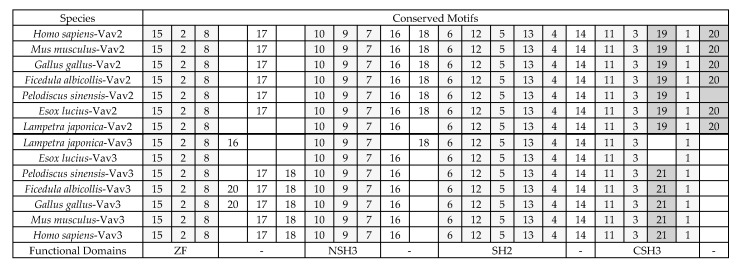
The distribution pattern of conserved motifs between Vav2 and Vav3 molecules. The conserved motifs (recurring, fixed-length patterns) were discovered with the online tool Multiple Em for Motif Elicitation (MEME) as described in Materials and Methods. The number and the widths of conserved motifs were set as 25 and 5–20 amino acids, respectively. The abbreviations of protein functional domains are the same as described in Figure 1. Light gray columns show the conserved motifs between Vav2 and Vav3 groups and dark gray columns mark the diversified motifs between Vav2 and Vav3 group. The protein accession numbers are included in Figure 1 and Figure 2.

To investigate the essential differences between Vav3 and Vav2 sequences, the distribution pattern of conserved motifs (recurring, fixed-length patterns) was analyzed by the online tool MEME with 14 sequences (lamprey Vav2, lamprey Vav3, six Vav3 molecules and six Vav2 molecules) from lamprey to mammals as described in Materials and Methods Section. Because the CH, Ac, DH and PH domains are highly conserved between Vav2 and Vav3 sequences (Figure 1), the conserved motifs were searched only among their ZF, NSH3, SH2 and CSH3 domains. Totally, 21 conserved motifs were found among these sequences (Table 1). The arrangements of 11 conserved motifs in ZF, NSH3 and SH2 domains are identical between Vav2 and Vav3 molecules (Figure 3). It is easy to find that the differences between Vav2 and Vav3 molecules are in their two CSH3 domains. Motif 19 (SKIGGDQ) and motif 20 (EEEGV) are the specific motifs of lamprey Vav2 and Vav2 molecules, while motif 21 (TKMSA) is the specific one of Vav3 molecules (Figure 3). The divergence on their CSH3 domains, which play important roles in substrates binding, indicated that Vav2 and Vav3 members diversified into two groups of functional independent genes through short deletion, insertion and substitution processes under the selection pressure of substrate specificity.

### 2.4. The Expression Pattern of Lamprey Vav3 mRNA and Protein after Antigen Stimulation

**Figure 4:**
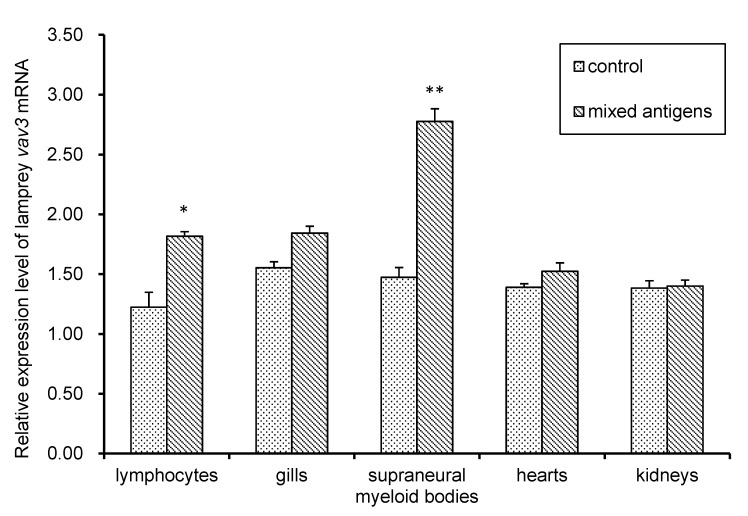
The relative expression levels of the lamprey *vav3* mRNA in immune-related tissues before and after challenged by mixed-antigens. The lamprey *vav3* mRNA levels were determined in immune-related tissues by using real-time quantitative PCR (Q-PCR) with an internal control, lamprey *gapdh*. The stimulated group was challenged with the mixed pathogens. The significant differences in lamprey *vav3* mRNA expression between the stimulated and the corresponding control groups are indicated with asterisks, *: *p* < 0.05, **: *p* < 0.01.

The real-time quantitative PCR (Q-PCR) was performed to investigate the expression pattern of lamprey *vav3* mRNA in immune-related tissues after antigen stimulation. As shown in Figure 4, lamprey *vav3* mRNA was ubiquitously expressed in lymphocytes, gills, supraneural myeloid bodies, kidneys and hearts in the control group. The strongest mRNA expression was detected in supraneural myeloid bodies. In the mixed-antigens-stimulated group, the relative expression level of lamprey *vav3* mRNA was significantly up-regulated in lymphocytes (*p* < 0.05). Moreover, the relative expression level of lamprey *vav3* mRNA in supraneural myeloid bodies was extremely significantly increased more than two folds in mixed-antigens-stimulated group relative to that of control group (*p* < 0.01). Although the relative expression level of lamprey *vav3* mRNA was obviously increased in gills after stimulation, the difference between the two groups was not significant (*p* > 0.05).

**Figure 5:**
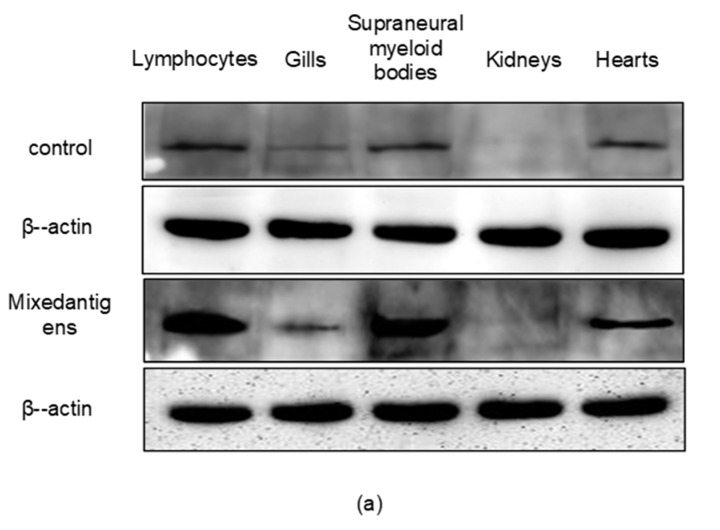

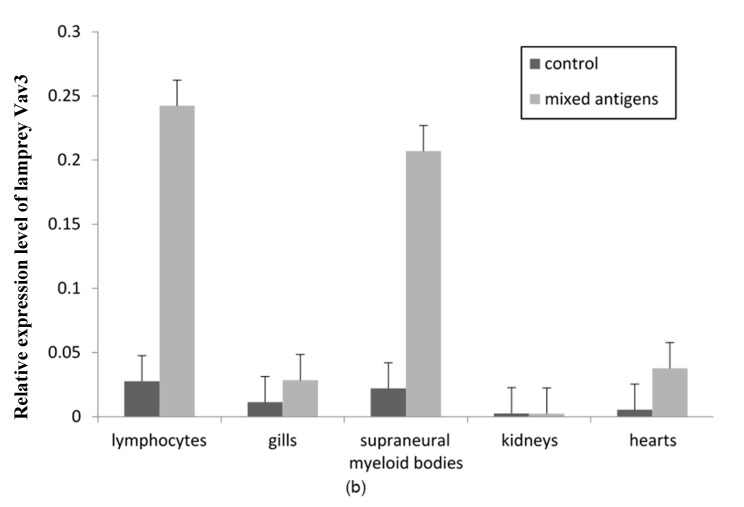
The relative expression levels of lamprey Vav3 in lamprey immune-related tissues after challenged with mixed-antigens. Total protein samples were isolated from immune-related tissues. (**a**) Western blotting analysis was performed to detect the expression levels of lamprey Vav3 with β-actin of *L. japonica* as an internal control; (**b**) A column chart created using data from three independent Western blotting analysis results. Data are presented as mean ± S.D. The significant difference *p* < 0.01 is shown with asterisks **.

Western blotting was performed with the anti-recombinant lamprey Vav3 polyclonal antibody (pAb) to detect the expression patterns of lamprey Vav3 in immune-related tissues after challenged by mixed-antigens. The internal control, β-actin of *L. japonica*, was detected as a band at 42-kDa (Figure 5a). The endogenous lamprey Vav3 was detected as a band at about 90-kDa in the samples of lymphocytes, supraneural myeloid bodies, hearts, gills and kidneys in control group. In the mixed-antigens-stimulated group, the relative expression levels of lamprey Vav3 in lymphocytes and supraneural myeloid bodies were about 10-fold more up-regulated than those of their corresponding control groupsy (Figure 5b). The fact that the relative expression levels of lamprey *vav3* mRNA and protein were significantly increased in lymphocytes and supraneural myeloid bodies after mixed-antigens stimulation indicates that lamprey Vav3 plays an important role in the immune response of lamprey lymphocytes.

**Figure 6:**
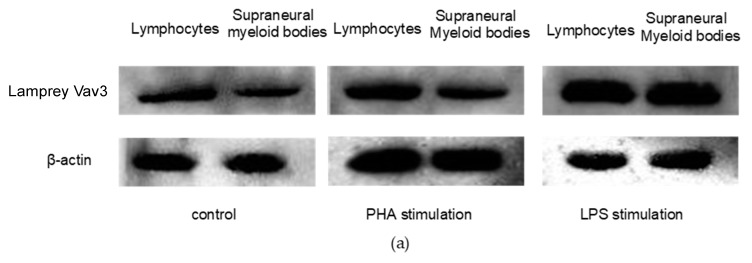

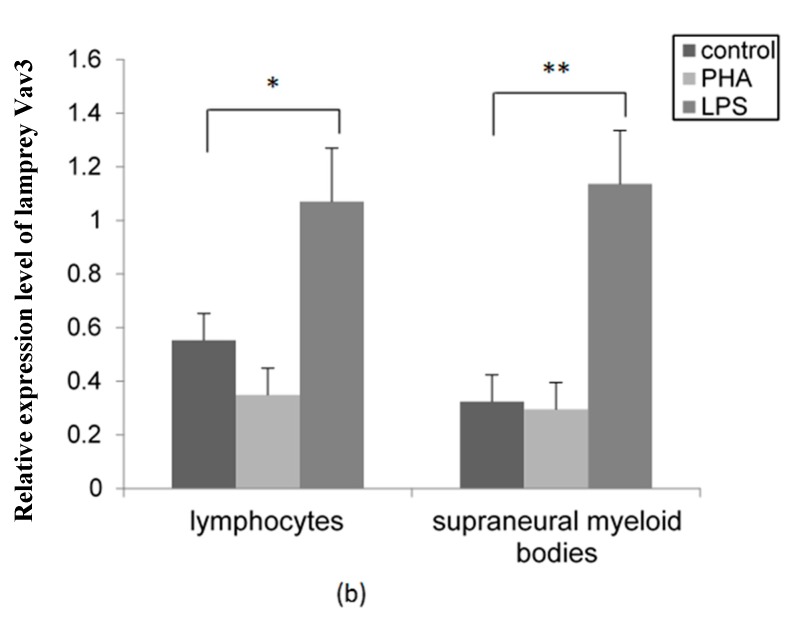
The expression property of lamprey Vav3 in response to the stimulation of lipopolysaccharide or phytohemagglutinin. Two groups of animals were stimulated with lipopolysaccharide (LPS) or phytohemagglutinin (PHA). Lymphocytes and supraneural myeloid bodies were isolated from the animals stimulated with LPS, PHA or not for extracting total protein samples. (**a**) Western blotting analysis was used to detect the expression levels of lamprey Vav3 as described in Materials and Methods Section; (**b**) A column chart created using data from three independent Western blotting analysis results. Data are presented as mean ± S.D. The significant differences *p* < 0.05 or *p* < 0.01 are shown with asterisks * or **, respectively.

The expression properties of lamprey Vav3 in response to the stimulation of lipopolysaccharide (LPS) and phytohemagglutinin (PHA) were also evaluated by Western blotting methods in tissues of lymphocytes and supraneural myeloid bodies. As shown in Figure 6, the relative expression levels of lamprey Vav3 in lymphocytes and supraneural myeloid bodies did not change much after PHA stimulation, but they were up-regulated 100% and 250% after 24-h LPS stimulation in lymphocytes and supraneural myeloid bodies, respectively. Our results reveal that lamprey Vav3 is involved in the LPS-mediated immune responses of lymphocytes and supraneural myeloid bodies.

### 2.5. The Distribution Pattern of Lamprey Vav3 in VLRB^+^ Lymphocytes after Stimulation with Lipopolysaccharide (LPS) and Phytohemagglutinin (PHA)

**Figure 7:**
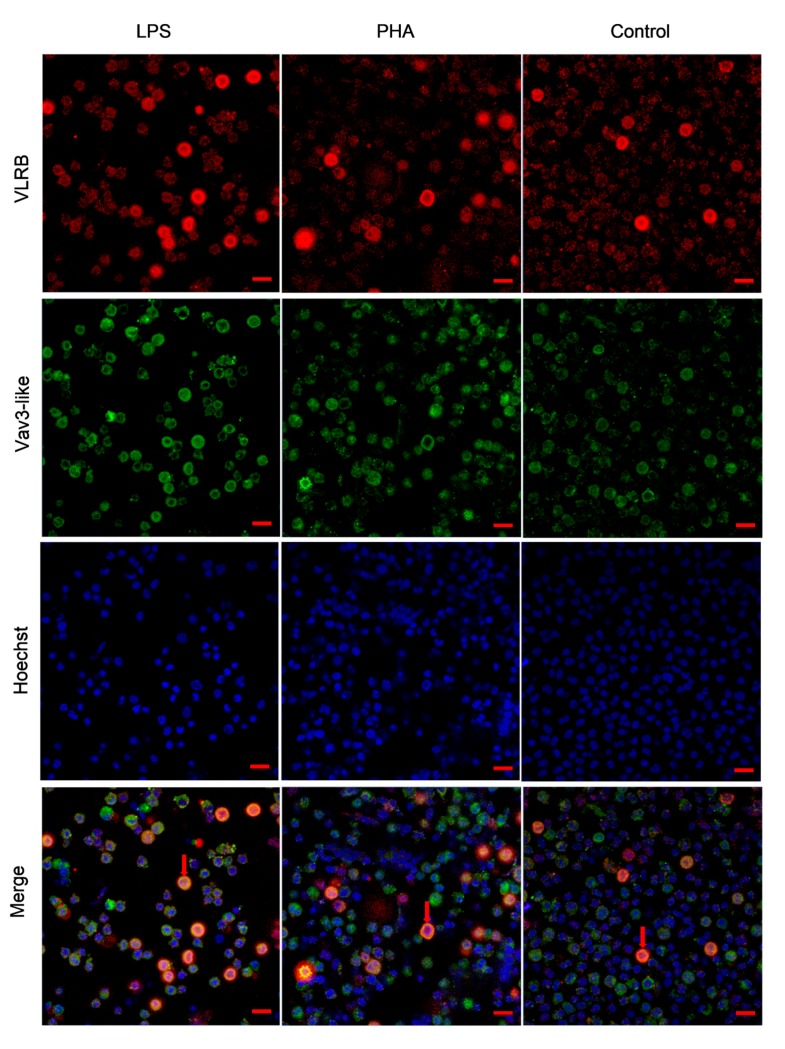
The distribution pattern of lamprey Vav3 in VLRB^+^ lymphocytes after stimulation with LPS and PHA. Lymphocytes were isolated from animals before and after treated with LPS or PHA. The cells were first incubated with the rabbit anti-recombinant lamprey Vav3 poly-clonal antibody and the mouse anti-VLRB mono-clonal antibody and then were incubated with Alexa Fluor 555-conjugated goat anti-mouse IgG (red) and Alexa Fluor 488-conjugated goat anti-rabbit IgG (green), as described in Materials and Methods Section. The nuclei were stained with Hoechst 33258 dye (blue). Cells were examined by a Zeiss LSM710 confocal laser scanning microscope. The bar represents 10 μm. The big and round cells (VLRB and lamprey Vav3 double positive) marked with arrows are plasmacytes.

To determine the distribution pattern of lamprey Vav3 in peripheral blood lymphocytes, an immunofluorescence assay was performed according to the description in Materials and Methods Section. As shown in Figure 7, the VLRB^+^ lymphocytes were stained in red color, and lamprey Vav3 (stained in green color) were detected with nearly all distributed in the cytoplasm of VLRB^+^ lymphocytes in all three groups. From merged photos, it is easy to see that the amount of some big and round VLRB and lamprey Vav3 positive lymphocytes in total VLRB^+^ lymphocytes were increased in LPS-stimulation groups (14.6%) compared to in control (2.9%) and PHA-stimulation (4.1%) groups according to the calculation method mentioned in Materials and Methods Section. According to the description of Alder et al. [28], these big and round VLRB^+^ lymphocytes are plasmacytes that can secrete multimeric antigen-specific VLRB antibodies. Thus, the increasing of effector VLRB^+^ cells further proved that lamprey Vav3 was involved in the LPS-mediated immune response of lamprey.

## 3. Discussion

The Vav family members, which play important roles in cell signaling and development, are a group of signaling molecules with oncogenic potential. The most typical function of Vav family members is to activate the nuclear factor of activated T-cells (NFAT) in lymphocytes, e.g., Vav2 and Vav3 can trigger NFAT activation only in B-cells but Vav1 can do so in both T- and B-cells [30,31]. It was found that the phosphorylation of Vav family members is mediated by some transmembrane or cytosolic protein tyrosine kinases such as Syk, Janus, Tec, Abl, and Src family kinases [11,32]. Although there is neither TCR nor BCR mediated adaptive immune system, lamprey lymphocytes were found not only to express homologous genes involved in some immunological activities relevant to mammalian lymphocytes [33], but also to express VLRs as the basis for antigen recognition [24]. Thus, it was proven that there is not only the innate immune system, but also an alternative adaptive immune system in jawless vertebrates [34]. Thus far, there are no reports on what constitutes the molecular basis of the signal transduction mechanisms of the lamprey lymphocytes. In this study, we have isolated and characterized a vertebrate Vav3 homologous molecule from *L. japonica* for the first time. The existence of this signaling molecule in lamprey lymphocytes makes us curious about the evolution of Vav family and the potential role it plays in lamprey.

To find other Vav homologs in lamprey, sequence alignment by BLAST tools in various databases was performed. Finally, only two Vav2 (accession number: gbAIN44441, ENSPMAT00000002100.1) and a Vav3 homologous sequences (accession number: ENSPMAT00000002350.1) were found in lamprey, and no Vav1 homologous sequence was found in various lamprey related databases. The existence of Vav2 and Vav3 homologs and the absence of Vav1 homolog in lampreys indicate that the ancestor gene of Vav family has split only once in lampreys through gene duplication event. The diversification of the lamprey Vav2 and Vav3 was further revealed by conserved motif analysis. As shown in Figure 3, their CSH3 domains diversified most. Lamprey Vav2 possesses motifs 19 and 20 which are well conserved among all Vav2 molecules in its CSH3 domain but absent in lamprey Vav3 and other Vav3 molecules. Lamprey Vav3 does not possess motif 21 which exist only in CSH3 domain of amniotes’ Vav3 molecules. Thus, it can be deduced that lamprey Vav2 is probably more primitive than lamprey Vav3 in evolution. This conclusion is supported by the result of phylogenetic analysis in a certain degree. Lamprey Vav2 and lamprey Vav3 are grouped together in the Vav2 cluster, and the genetic distance between the common ancestors of Vav2 subfamily and Vav family is the shortest, indicating that the Vav2 subfamily originated earlier than Vav3 and Vav1 subfamilies.

The similar structure between lamprey Vav3 and mammalian Vav3 implied that they may have similar functions (Figure 1). Lamprey *vav3* was proven to be transcribed and distributed widely in several immune-related tissues, and this widely distribution pattern is in accordance with that of vertebrate *vav3* [2,3]. Lamprey *vav3* mRNA was found significantly up-regulated in the lymphocytes, and extremely up-regulated in supraneural myeloid bodies after stimulated by mixed antigens (Figure 4). It was further confirmed by Western blot analysis that the relative expression level of lamprey Vav3 in the mixed-antigens-stimulated group was also up-regulated in the lymphocytes and supraneural myeloid bodies (Figure 5). The above results showed that the lamprey Vav3 is functionally involved in the immune response of lamprey. Further deep investigations, such as the binding and function of lamprey Vav3 to RhoA, RhoG and Rac1, are needed to clarify the precise role of lamprey Vav3 played in immune response.

New evidence suggested that the similar organizations of thymus are discovered in the gill filament tips of lampreys, and VLRA^+^ cells develop in this thymoid region. In some functional regards, VLRA^+^ lymphocytes can be proliferated as T cells after PHA (a kind of T cell mitogen) stimulation [25,35]. In the present study, PHA and LPS (a kind of B cell mitogen) were also used to examine the lamprey Vav3 expression property. Intriguingly, the relative expression levels of lamprey Vav3 neither changed obviously in lymphocytes and supraneural myeloid bodies after PHA stimulation (Figure 6), nor changed in gills after mixed-antigen stimulation (Figure 5). In addition, the number of plasmacytes was not changed obviously in VLRB and lamprey Vav3 double positive lymphocytes after PHA stimulation (Figure 7). This suggests that lamprey Vav3 may not be involved in the PHA-mediated immune response of lamprey, but further studies are still needed to verify the exact relationship of lamprey Vav3 with VLRA^+^ or VLRC^+^ lymphocytes.

Recently, Btk, Blnk and Syk homologs which are involved in vertebrates’ BCR signaling pathway have also been identified in lamprey [36,37,38,39]. They were found un-regulated in lymphocytes and supraneural myeloid bodies after LPS or mixed-antigens stimulation. In the current study, we also found that lamprey Vav3 distribute in VLRB^+^ lymphocytes and are up-regulated in the lymphocytes and supraneural myeloid bodies after LPS or mixed-antigens stimulation. Amemiya et al. [23] found that lamprey supraneural myeloid body presents every type of blood cells in all stages of maturity including their precursors after hematopoietical stimulation, a property seems to be highly similar to “bone marrow” in higher vertebrates. The consistent reaction of these signaling molecules (Syk, Btk, Blnk and lamprey Vav3) in lamprey lymphocytes and supraneural myeloid bodies indicated that they may play important roles in the immune response of lamprey VLRB^+^ lymphocytes (effector B-like cells). These suggest that although the transmembrane adaptor of VLRB is still unknown, VLRB and BCR have conserved molecular basis in their intracellular signaling pathways.

## 4. Materials and Methods 

### 4.1. Handing of Animals

The care of laboratory animal and the animal experimental operation have conformed to the guidelines of *Administration Rule of Laboratory Animal* of Chinese government and were permitted by the Liaoning Normal University Animal Welfare and Research Ethics Committee (issued on 6 March 2011). Lampreys were bought from a market of Tongjiang Valley (Jiamusi City, Heilongjiang, China) and kept in laboratory aquarium which equipped with a physical and biological filtration system under 4 °C. The animals in the stimulated and control groups were intraperitoneal injected with 100 µL antigens or normal saline, respectively. In the current study, three kinds of antigens (the mixed-antigens, lipopolysaccharide (LPS, Sigma-Aldrich Co. LLC, Saint Louis, MO, USA, 30 μg/100 μL in normal saline) and phytohemagglutinin (PHA, Sigma-Aldrich, 30 μg/100 μL in normal saline)) were used to immune lampreys. The mixed-antigens contained equal amount heat-inactive three microbial strains (1 × 10^7^ cfu/mL, in normal saline) including the representatives of gram-negative (*Escherichia coli* DH5α), gram-positive (*Staphylococcus aureaus*) and fungi (*Saccharomyces cerevisiae*). The animals were immunized twice at 7-day intervals and were sacrificed for taking tissues 24 h after the second immunization [40].

### 4.2. Amplification of the Lamprey vav3 cDNA Fragment

The lymphocyte-like cells were isolated from peripheral blood by using Ficoll density gradient centrifugation method as described by Liu et al. [41]. Total RNAs were extracted from lymphocyte-like cells of *L. japonica* by using RNAiso reagent bought from TaKaRa Biotechnology (Kusatsu, Japan) CO., LTD. (Dalian, China) and kept in RNase-free water. Total RNA samples were reverse transcribed to cDNA sequences by using PrimeScriptTM II First Strand cDNA Synthesis Kit (TaKaRa Biotechnology). The ORF of lamprey *vav3* was amplified by using PCR method with a pair of sense and antisense primers listed in Table A1.

### 4.3. Real-Time Quantitative PCR

Total RNA samples were isolated separately from lymphocytes, supraneural myeloid bodies, gills, kidneys and hearts. The supraneural myeloid bodies were isolated according to the description of George et al. [42]. The cDNA sequences were synthesized from total RNA samples by reverse transcription with PrimeScript™ RT Reagent Kit (TaKaRa Biotechnology). The TaKaRa SYBR^®^ PrimeScript™ RT-PCR Kit was used to perform Q-PCR according to the manufacturer’s instruction. The starting quantity of RNA was normalized by the internal control, the *gapdh* of *L. japonica* (accession number KU041137). The primers for amplifying *gapdh* and lamprey *vav3* cDNA fragments were listed in Table A1. The cycling system was the same as described by Zhang et al. [43]. Triplicate experiments for each sample were carried out and the results were shown as mean ± standard deviation (S.D.).

### 4.4. Analyses of Sequence Similarity and Conserved Motifs and Phylogenetic Tree Reconstruction

The protein sequences of Vav3, Vav2 and Vav1 molecules were searched from NCBI database (Available online: http://www.ncbi.nlm.nih.gov/) to conduct sequence similarity, conserved motifs and phylogenetic analyses. Multiple sequence alignments of Vav-like with some jawed vertebrates Vav3 and Vav2 molecules were conducted using BioEdit 7.0 software (Micro Focus, Newbury, United Kingdom). The protein conserved domains were identified by the Simple Modular Architecture Research Tool (SMART, available online: http://smart.embl-heidelberg.de/). The phylogenetic tree was reconstructed by software MEGA 4.0 (Available online: http://www.megasoftware.net/mega4/)with neighbor-joining (NJ) method [37]. The conserved motifs (recurring, fixed-length patterns) were discovered by the Multiple Em for Motif Elicitation tool (MEME, available online: http://meme-suite.org/tools/meme) [44]. The number and the widths of conserved motifs were set to 25 and 5–20 amino acids, respectively.

### 4.5. The Expression and Purification of Rlamprey Vav3

The expression and purification of recombinant lamprey Vav3 were conducted by following the description of Han et al. [38]. Briefly, the ORF region of lamprey *vav3* was sub-cloned into the expression vector pET-32a (+) by introduction of two restriction sites (*EcoR* I and *Hind* III) as ligation sites. The recombinant lamprey Vav3 was overexpressed in *E. coli* BL21 (DE3), and the insoluble fraction of inclusion body was collected by centrifugation, and the pellet was dissolved in 6M urea solution and purified with Ni affinity chromatography (GE Healthcare, New York, NY, USA). The purified recombinant lamprey Vav3 was concentrated to about 0.5 mg/mL by dialysis against 20% polyethylene glycol 6000 solution (Sangon Biotech, Shanghai, China) and stored at −20 °C.

### 4.6. Mass Spectrometry of Recombinant Lamprey Vav3 Protein

Recombinant lamprey Vav3 protein was identified by peptide mass fingerprinting technique with an autoflex™ speed MALDI-TOF mass spectrometer (Bruker Daltonics Inc., Billerica, MA, USA). The purified recombinant lamprey Vav3 was analyzed by 12% sodium dodecylsulfate -polyacrylamide gel electrophoresis (SDS-PAGE). The bands were excised from coomassie stained SDS-PAGE gel and digested with sequencing grade modified trypsin (Catalog No: V5111, Promega Corporation, Madison, WI, USA) after removal of coomassie staining. The digested peptides in the gel slices were extracted by the method described by Shevchenko et al [45]. The matrix-assisted laser desorption/ ionization time of flight (MALDI-TOF) mass spectrometry was operated in the positive ion mode with the following acquisition cycle: a full scan (*m*/*z* 750) recorded in the orbitrap analyzer at resolution R 60,000 and then followed by MS/MS of the 20 most intense peptide ions in the LTQ analyzer. All MS raw data were searched against all lamprey sequences available in the NCBI database using the MS-Mascot searching algorithm. Search criteria used were as follows: oxidation of Met, carbamidomethylation of Cys, Trypsin, 0.5 Da peptide mass to tolerance, 1 Max missed cleavage sequence coverage >10%.

### 4.7. Production of Polyclonal Antibody

The rabbit anti-recombinant lamprey Vav3 polyclonal antibody (pAb) was generated according to the description of Han et al. [38]. The titer of the pAb against recombinant lamprey Vav3 was checked by enzyme-linked immunosorbent assay (ELISA). The pAb was verified using Western blot method with purified recombinant lamprey Vav3 as standard protein samples. The pAb was purified by chromatography method with a CNBr-activated sepharose 4B column (GE Healthcare) and stored at −20 °C.

### 4.8. Western Blotting Analysis

Total protein samples were extracted from five immune-related tissues mentioned above with a cell lysis buffer (Beyotime, Beijing, China). The cell debris was removed from lysate by centrifugation at 12,000 rpm for 15 min at 4 °C. Total protein samples or the purified recombinant lamprey Vav3 were separated by 12% SDS-PAGE, and then transferred on the polyvinylidene fluoride (PVDF) membranes. The following procedures of Western blotting were done according to the description of Li et al. [40]. The signal intensity data were obtained and calculated from three independent experiments.

### 4.9. Immunofluorescence Assay

Lymphocyte cells were isolated from animals before and after treated with LPS or PHA, and were suspended in 1.5 mL Eppendorf tubes and fixed with paraformaldehyde solution (4% in phosphate buffer saline (PBS)) for 20 min at room temperature. Then, Immunofluorescence assay was performed according to the description of Han et al. [38] with a rabbit anti-recombinant lamprey Vav3 poly-clonal antibody (1000-fold) and a mouse anti-VLRB mono-clonal antibody (1000-fold) [46]. Cells were observed by a Zeiss LSM710 Confocal Laser Scanning Microscope (Oberkochen, Germany) and each type of cells (including big and round VLRB^+^ plasmacytes and small VLRB^+^ lymphocytes) were counted and analyzed in 5 microscope fields by using Zeiss ZEN LE software.

### 4.10. Statistical Analysis

Data were shown as mean ± S.D. The significance of the difference between two groups was evaluated by SPSS statistical software package with a Student’s *t* test. Differences were considered statistically significant or extreme significant at *p* < 0.05 or *p* < 0.01, respectively.

## 5. Conclusions

In conclusion, a Vav3 homolog was characterized in lamprey; the divergence of lamprey Vav2 and lamprey Vav3, the only two members of Vav family identified in lampreys, is in their CSH3 domains. Lamprey *vav3* mRNA and protein were significantly up-regulated in lymphocytes and supraneural myeloid bodies after mixed-antigens and LPS stimulation, indicating that lamprey Vav3 should be involved in the LPS-mediated immune response of lamprey. The distribution of lamprey Vav3 in the cytoplasm of VLRB^+^ lymphocytes and the increased number of effector VLRB^+^ lymphocytes after LPS stimulation provided a clue for the further study of the precise role of lamprey Vav3 played in the signaling pathway of lamprey VLRB^+^ lymphocytes.

## Figures and Tables

**Table 1:** Conserved motifs discovered between Vav3 and Vav2 molecules from different species using the MEME software.

Motifs	Width	Best Possible Match	Motifs	Width	Best Possible Match
1	20	QGWWKGEVNGRVGWFPSTYV	2	20	TNCKACQMLLRGTFYQGYLC
3	20	FCARDMRELSLREGDVVKIY	4	20	LVEYYQHHSLKEGFRQLDTT
5	20	YAISIKFNNEVKHIKIVTKD	6	20	DYSAYPWFAGNMERQQADNE
7	20	WWQGRNLQTQKVGYFPSDAV	8	20	SKCGAGAHKECLEIIDNCKM
9	20	PPLHFQTGDVIELLRGDPHS	10	20	DPGLPKMQAIQNYHGIPAPP
11	20	RTRSPVFTPRVIGIAIARYD	12	20	LINHVNGTYLIRHRTAEAEE
13	16	NWFHITENKKFKSLME	14	12	LQYPYKERENST
15	9	HNFQMHTFD	16	9	KPCPCDPKP
17	9	HPHDMDTNG	18	7	IRPPSRE
19	7	SKIGGDQ	20	5	EEEGV
21	5	TKMSA			

**Figure A1:**
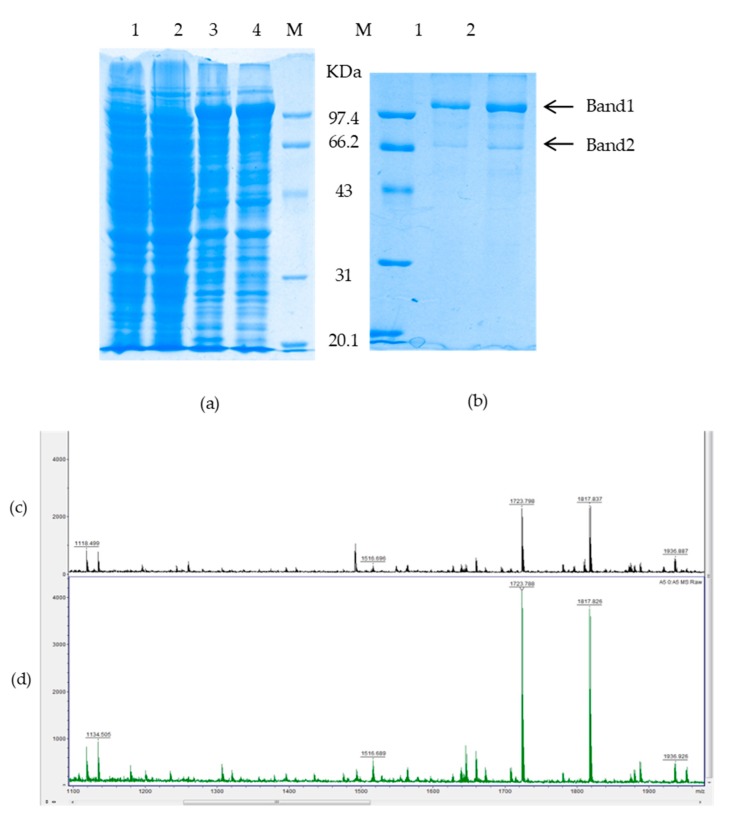
The expression, purification and identification of recombinant lamprey Vav3 his-tag fusion protein. A pET-32a (+) vector was used as the expression vector for expressing lamprey Vav3 open reading frame (ORF) region in *Escherichia coli* BL21 (DE3) as a fusion protein with 6× His-tag. The recombinant lamprey Vav3 was separated by 12% SDS-PAGE and stained with coomassie brilliant blue R-250. (**a**) The recombinant lamprey Vav3 was overexpressed in *E. coli* BL21 (DE3) strain by 1 and 0.1 mM IPTG induction. M: protein marker; 1: crude lysate of *E. Coli* BL21 (DE3) transformed by pET-32a (+) vector with 1mM IPTG induction; 2: crude lysate of *E. Coli* BL21 transformed by pET-32a (+lamprey Vav3) vector without IPTG induction; 3: crude lysate of *E. Coli* BL21 transformed by pET-32a (+lamprey Vav3) vector with 1mM IPTG induction; 4: crude lysate of *E. Coli* BL21 transformed by pET-32a (+lamprey Vav3) vector with 0.1mM IPTG induction; (**b**) The recombinant lamprey Vav3 his-tag fusion protein was purified by using affinity chromatography method with Ni-NTA His-Bind^®^ Resin. M: protein marker; 1: target protein eluted with 25 mM imidazole elution buffer; 2: target protein eluted by 100 mM imidazole elution buffer; (**c**,**d**) Peptide mass fingerprinting analysis of recombinant lamprey Vav3 protein bands 1 and 2 by an autoflex™ speed MALDI-TOF mass spectrometer.

**Figure A2:**
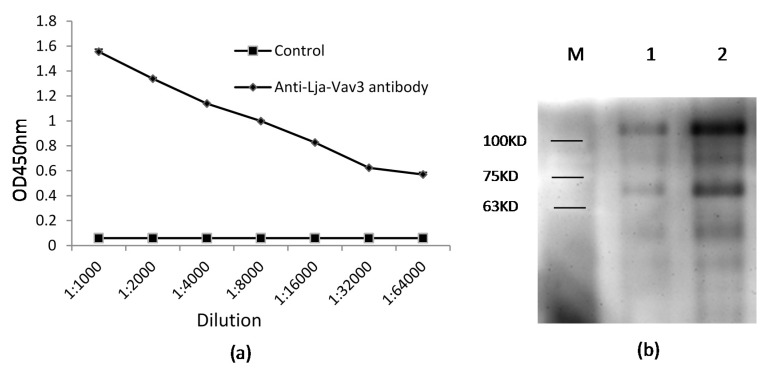
Titer and specificity analysis of the pAb against recombinant lamprey Vav3. (**a**) Determination of the titer of anti-recombinant lamprey Vav3 pAb by ELISA method. The anti-recombinant lamprey Vav3 pAb and negative control (rabbit IgG) were serially diluted to 64,000-fold in the same way, and the signal of each dilution was detected by three independent experiments; (**b**) the specificity of the anti-recombinant lamprey Vav3 pAb was confirmed by Western blot method. M: molecular marker; lane 1: the purified recombinant lamprey Vav3 (5 ng); lane 2: the purified recombinant lamprey Vav3 (15 ng) (*n* = 3).

## Abbreviations

Vav	Vav guanine nucleotide exchange factor
TCR	T-cell receptor
BCR	B-cell receptor
LPS	Lipopolysaccharide
PHA	Phytohemagglutinin
SH	Src-homology
VLR	Variable lymphocyte receptor
ORF	Open reading frame
NJ	Neighbor-Jointing
MEME	Multiple Em for Motif Elicitation
Q-PCR	The real-time quantitative PCR
pAb	Polyclonal antibody
NFAT	The nuclear factor of activated T-cells
SMART	Simple Modular Architecture Research Tool
ELISA	Enzyme-linked immunosorbent assay
PVDF	Polyvinylidene fluoride
IPTG	Isopropyl β-d-1-thiogalactopyranoside

## Appendix A. The Expression and Purification of the Recombinant Lamprey Vav3 Protein

The ORF region of lamprey Vav3 was amplified with expression primers, which contained *EcoR* I and *Hind* III restriction sites listed in Table A1. The amplified cDNA fragment was ligated with pET-32a (+) plasmid after double digestion with *EcoR* I and *Hind* III endonucleases. The recombinant expression vector was successfully transformed into *E. coli* BL21 (DE3) strain. The recombinant lamprey Vav3 was overexpressed by induction with 1 or 0.1 mM Isopropyl β-d-1-thiogalactopyranoside (IPTG) as a His-tag fused inclusion body protein (Figure A1a). After purification with Ni^2+^-affinity chromatography, the SDS-PAGE was used to check the purity of the product. There are two bands on the gel, a 120-kDa band that is in accordance with the recombinant lamprey Vav3 molecular mass, and a weak 67-kDa band (Figure A1B). The peptide mass fingerprint of the 67-kDa band (Figure A1C) was identified the same as that of the 120-kDa band (Figure A1D) by MALDI-TOF mass spectrometry analysis with significant protein scores (*p* < 0.05). Both peptide peaks at *m*/*z* 1817.837 were identified as R.AVQDDSQVFELAQVLR.D, which is a partial sequence of the lamprey Vav3 (Figure 1). The purified recombinant lamprey Vav3 was adjusted to about 0.5 mg/mL for antibody generation.

**Table A1:** The sequences of primers used in this study.

Name	Sequences
Primers designed for Lja-Vav3 open reading frame (ORF) cloning	
Forward-Lja-vav3	5′-ATGGAGGAAGAGGGCAGGTT-3′
Reverse-Lja-vav3	5′-CGTGGAAGAAGAAATGCTCTGA-3′
Primers designed for real-time PCR	
Lja-vav3 (upstream)	5′-ACCTGCGTCAACAGATTCGG-3′
Lja-vav3 (downstream)	5′-CACCGATGCCTTTTTTCTGC-3′
gapdh (upstream)	5′-ACCAACTGCCTGGCTCCT-3′
gapdh(downstream)	5′-TCTTCTGCGTTGCCGTGT-3′
Primers for subcloning Lja-vav3 ORF into the pET-32a vector	
Upstream	5′-ATGCCTGATATCGGATCCGAATTCATGGAGGAAGAGGGCAGGTTGTGGC-3′
Downstream	5′-GTGCTCGAGTGCGGCCGCAAGCTTGAGCATTTCTTCTTCCACGTAGGAG-3′

## Appendix B. The Production and Verification of Polyclonal Antibody

The pAb against recombinant lamprey Vav3 was generated as described in Materials and Methods Section. The titer of rabbit anti-recombinant lamprey Vav3 pAb was checked by ELISA method and it was higher than 1:32,000 (Figure A2a). The pAb was then purified by affinity chromatography method from rabbit antiserum and its specificity against recombinant lamprey Vav3 was verified by Western blot method. Two bands could be detected by the pAb, one 120-kDa band corresponding to the full-length recombinant lamprey Vav3, and another 67-kDa band corresponding to a partial synthesized recombinant lamprey Vav3 (Figure A2b).
